# The First Reported Case of Ocular Syphilis in an Iranian Patient

**DOI:** 10.18502/jovr.v18i4.14559

**Published:** 2023-11-30

**Authors:** Sahba Fekri, Shahram Salehi-Rad, Hosein Nouri, Shabnam Tehrani, Bita Shalbafan, Seyed-Hossein Abtahi

**Affiliations:** ^1^Ophthalmic Research Center, Research Institute for Ophthalmology and Vision Science, Shahid Beheshti University of Medical Sciences, Tehran, Iran; ^2^Labbafinejad Medical Center, Shahid Beheshti University of Medical Sciences, Tehran, Iran; ^3^School of Medicine, Isfahan University of Medical Sciences, Isfahan, Iran; ^4^Infectious Diseases and Tropical Medicine Research Center, Shahid Beheshti University of Medical Sciences, Tehran, Iran; ^5^Clinical Research Development Center of Labbafinejad Hospital, Shahid Beheshti University of Medical Sciences, Tehran, Iran; ^7^Sahba Fekri, https://orcid.org/0000-0002-7388-6725; ^8^Hosein Nouri, https://orcid.org/0000-0003-1808-0443

**Keywords:** Syphilis, Neurosyphilis, Bacterial Ocular Infection, Uveitis

## Abstract

**Purpose:**

To report the first case of ocular syphilis in an Iranian patient and discuss its diagnostic challenges.

**Case Report:**

A man in his mid-70s presented with progressive bilateral visual and auditory decline. He had previously lived in a Southeast Asian country for 10 years. Prior steroid therapies entailed no inflammation subsidence. His visual acuity at presentation was light perception OU. Funduscopic findings included severe vitritis, severe optic atrophy, diffuse retinal vascular occlusion, and diffuse retinal atrophy OU. Angiography demonstrated diffuse areas of retinal and choriocapillaris atrophy with no active choroiditis. Scaly cutaneous lesions were noted on his palms and soles – atypical findings of secondary syphilis. Serum analysis revealed an underlying syphilis infection. The cerebrospinal fluid sample was reactive to anti-syphilis antibodies, securing a neurosyphilis diagnosis. Two weeks of antibiotic therapy resulted in cutaneous lesions resolution and relative visual improvement despite extensive baseline retinal atrophic damage.

**Conclusion:**

Ocular syphilis can mimic numerous ocular inflammatory scenarios. In cases of ocular inflammation that is unresponsive to steroids, reconsidering alternative diagnoses, especially infections with the highest clinical relevance, is necessary. We stress the importance of acquiring patients' sexual history, regardless of cultural barriers and the rarity of the entity in some regions.

##  Introduction

Syphilis is caused by the *Treponema Pallidum* spirochete, which is transmitted most commonly through sexual contact. The global prevalence of syphilis in 2016 was estimated at around 0.5% for both genders, for a total of 5.5–7.1 million cases^[1]^. The clinical features are not specific and vary with different chronological stages of the infection – i.e., primary, secondary, tertiary, and latent. Neurosyphilis is caused by the invasion of the central nervous system (CNS) by the bacterium, it can occur at any stage and encompasses ocular and auditory syphilis as well^[2]^.

Ocular syphilis may develop in less than one percent of cases^[3]^; nearly all ocular structures may be affected by the infection and its consequent inflammation during primary, secondary, latent, or tertiary syphilis. Patients may present with a wide variety of symptoms, such as eye redness, vision impairment/loss (with or without pain), and photopsia^[2]^. Ocular syphilis can occur as early as six weeks after the primary infection, and in some cases, it may be the first manifestation of systemic infection^[4]^. When encountering eyes with various inflammatory presentations, it is crucial to include ocular syphilis among the differentials. However, its rarity in some regions, including Iran, may lead to its underdiagnosis and underreporting.

To our knowledge, ocular syphilis has not been reported in Iran. Here, we describe its first case in an Iranian patient, which manifested as painless gradual vision loss and led to near-complete blindness due to delayed diagnosis and treatment.

**Figure 1:**
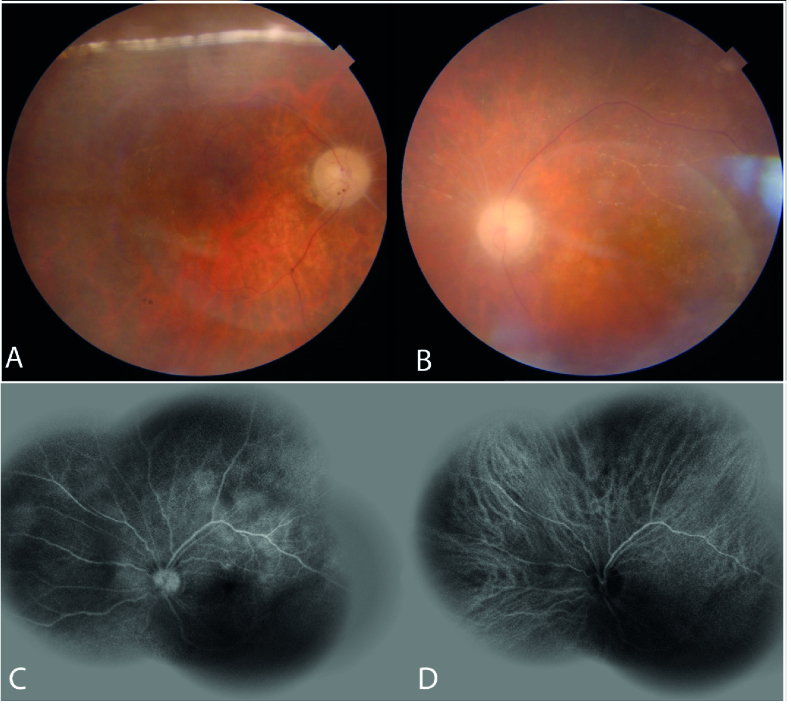
Fundus photography showing binocular blurred images and faint disc boundary, optic disc atrophy, multiple sclerotic occluded vessels – predominantly affecting the arteries rather than veins, severe ischemia, and punctate retinitis (A and B; OD and OS); pre-treatment fluorescein angiography and indocyanine green angiography (FA+ICGA) of the left eye showing diffuse, non-specific areas of staining, venous wall staining, and delayed arterial perfusion (C and D; OS).

**Figure 2:**
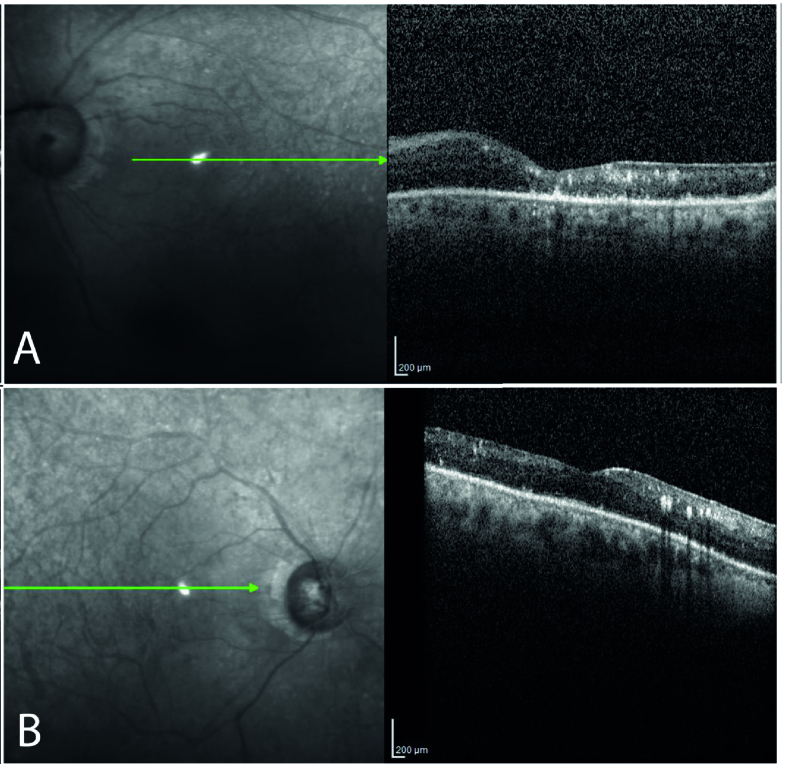
B-scan structural optical coherence tomography showing diffuse atrophy in the entire inner and outer retinal layers, disruption of the ellipsoid zone and choriocapillaris, and multiple foci of retinitis (hyper-reflective dots) (A, OS; B, OD).

**Figure 3:**
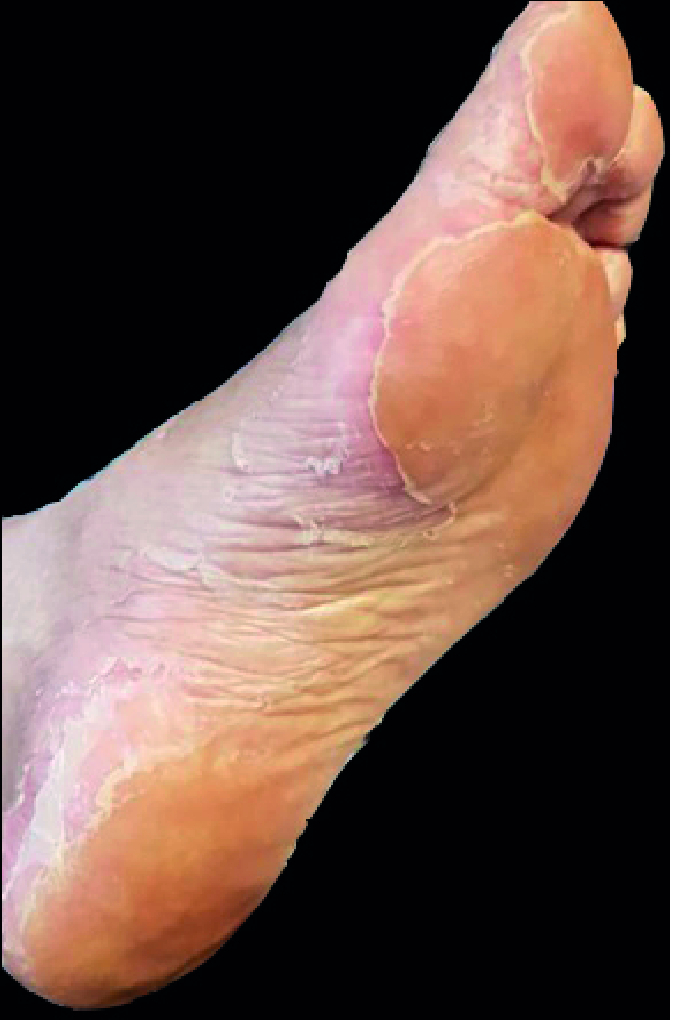
Plantar cutaneous lesions; lesions had appeared in temporal proximity of the visual symptoms onset.

**Figure 4:**
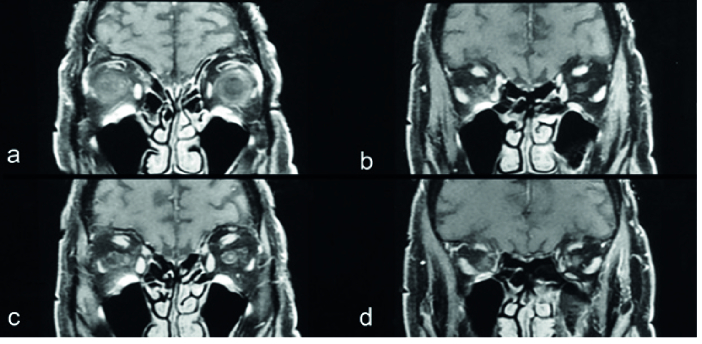
Coronal T1-weighted brain MRI scans with gadolinium administration showing mild optic nerve enhancement, visualizing no brain lesions or intraocular tumors.

**Figure 5:**
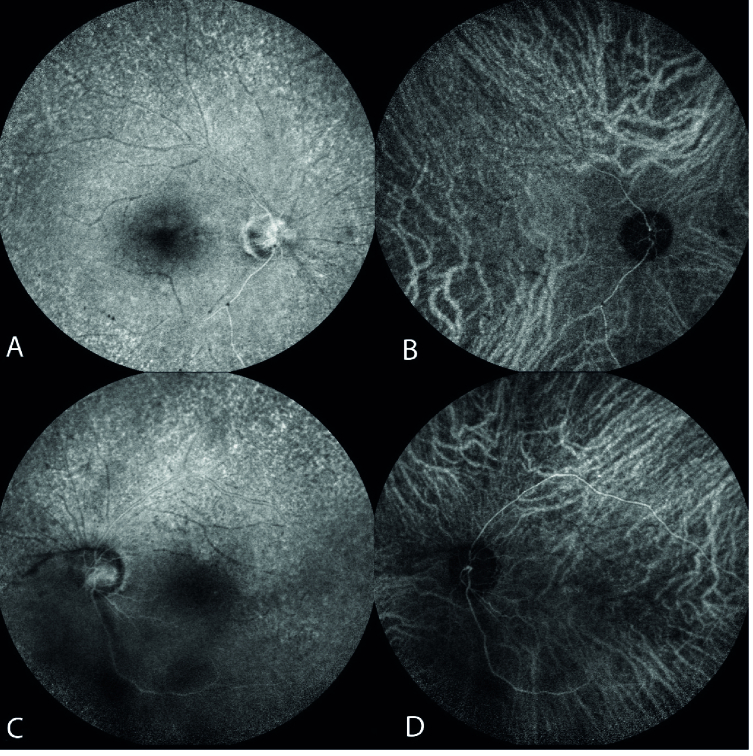
Post-treatment fluorescein angiography and indocyanine green angiography (FA+ICGA), demonstrating diffuse chorioretinal atrophy, severe narrowing of arteries, and delayed vessel perfusion (A and B, OD; C and D, OS).

##  Case report

A 75-year-old man presented with a three-month history of progressive bilateral vision and hearing decline. His social history was significant for a previous divorce and then living for more than 10 years in Thailand. Three months before his admission to our center, his symptoms had appeared as visual floaters and mild bilateral visual and auditory impairment, for which he had visited several ophthalmologists. His visual symptoms continuously aggravated throughout those three months until he visited our center.

Upon his presentation, he had already been diagnosed with intermediate uveitis by an ophthalmologist who administered high-dose oral steroids for three weeks along with multiple trans-septal steroid injections. This prescribed treatment however, resulted in no subsequent improvement; instead, it led to further gradual deterioration of visual and auditory senses. Given the simultaneous loss of vision and hearing, multiple brain magnetic resonance imaging (MRI) sessions had been performed to rule out intraocular lymphoma. The patient was then referred to our department for further evaluation.

The patient did not report any genital chancre or regional lymphadenopathy; he also denied its existence when confronted. In our examination, his visual acuity was light perception (OU). Upon biomicroscopic examination, 2+ cells were detected in the anterior chamber of both eyes. Pupils were round and showed sluggish reactions without notable irregularities or relative afferent pupillary defects. The dilated fundus examination revealed the following: a) hazy media due to severe vitritis, b) severe optic atrophy, c) diffuse retinal vascular occlusion – due to occlusive retinal arteriolitis, and d) diffuse retinal atrophy – in both eyes (Figure 1. A & B). Complete uveitis workup and diagnostic vitrectomy were performed to rule out possible malignant or infectious pathologies. We were then able to carry out simultaneous fluorescein angiography and indocyanine green angiography (FA+ICGA) for suspected concurrent involvement of the choroid in the left eye – which had undergone vitrectomy. FA+ICGA demonstrated retinal involvement and diffuse atrophy of choriocapillaris, but no apparent sign of choroidal stromal involvement or vasculitis (Figure 1. C & D). Optical coherence tomography revealed multiple foci of retinitis, diffuse retinal atrophy, affecting the inner and outer retina, with disruption of the ellipsoid zone and choriocapillaris (Figure 2). The patient had moderately itchy, scaly cutaneous lesions localized at his palms and soles (Figure 3). He mentioned the simultaneity of skin lesions appearance with the onset of his visual decline. Vitreous cytologic analyses (Papanicolaou and Diff-Quick stains) and infectious antigen investigations (herpes simplex virus, varicella-zoster virus, cytomegalovirus, toxoplasma gondii, and tuberculosis) were all negative. HIV-specific antibodies were not detected in his serum via enzyme-linked immunosorbent assay (ELISA). The Venereal Disease Research Laboratory (VDRL) blood test was reactive, and a subsequent positive fluorescent treponemal antibody-absorption (FTA-abs) test result confirmed a syphilis infection.

Consultations with neurology and infectious services were done, and the patient underwent brain imaging and lumbar puncture for cerebrospinal fluid (CSF) sampling. CSF FTA-abs Immunoglobulin-G/Immunoglobulin-M (IgG/IgM) and electrophoresis of oligoclonal bands for IgG were positive. CSF pleocytosis and increased protein levels were noted. Brain MRI scans with gadolinium showed mild optic nerve enhancement without any apparent brain lesions (Figure 4).

With the diagnosis of neurosyphilis, the patient underwent treatment with ceftriaxone (2 g/day, intravenously for 14 days), and his steroid therapy was continued with a maintaining dose of 7.5 mg/day oral prednisolone. One month after the treatment, the patient's visual acuity improved to counting fingers at one meter (OU). Post-treatment FA+ICGA was performed for both eyes which showed diffuse chorioretinal atrophy, severe arterial narrowing, and perfusion delay (Figure 5). After the treatment, no sign of his atypical plantar or palmar cutaneous lesions was present. The patient's consent was obtained for publication of his case subject to anonymization.

##  Discussion

Depending on the affected part of the eye, ocular syphilis may manifest through several clinical patterns. Among those inflammatory patterns, uveitis is the most common form. The inflammation most frequently affects the entire uvea (i.e., panuveitis), and less frequently results in anterior, intermediate, or posterior uveitis. Other clinical patterns that may be present in cases of ocular syphilis include scleritis/episcleritis, optic neuritis, conjunctivitis, blepharitis, tarsitis, and eyelid chancre – i.e., when the eyelid has been the site of primary infection^[2]^. Although some patients report a history of syphilis infection, many notice it for the first time when they have developed ocular complications^[5]^. The estimated prevalence of syphilis in Iran does not exceed 1%; most cases are from high-risk groups, such as sex workers and HIV-positive people^[6]^. Given its rarity in Iran and its overlapping clinical features, the most crucial element in a timely diagnosis of ocular syphilis is having a strong suspicion, for which a complete understanding of the history of social and sexual status is necessary. Unfortunately, some cultural barriers may impede the acquisition of an adequately-detailed sexual history – in many instances, both patients and physicians may find it difficult to share or ask for such information.

When syphilis is suspected, serological screening and confirmatory tests for non-treponemal (e.g., VDRL) and treponemal antibodies (e.g., FTA-abs), respectively, should be performed^[7]^. The Center for Disease Control and Prevention recommends CSF analysis in all persons diagnosed with syphilis who have neurologic, auditory, and ophthalmic abnormalities. The CSF analyses constitute CSF-VDRL and CSF FTA-abs. The former is highly specific but insensitive, while the latter has higher sensitivity but less specificity for neurosyphilis. A reactive CSF-VDRL secures a diagnosis of neurosyphilis. CSF leukocytosis and elevated protein levels can help with the diagnosis when the mentioned antibody test results are indefinite^[8]^.

We assume that as a result of the presence of the following factors together: i) the rarity of ocular syphilis in Iran, ii) cultural hurdles to asking about patients' sexual habits, and iii) the pattern of ocular involvement, previous ophthalmologists had put intraocular lymphoma at the top of their suspicion list, while ocular syphilis work up had not been performed. Unfortunately, this led to delayed diagnosis confirmation, improper treatment regimens, and permanent damage to both eyes.

Another key message of this case is that when ocular inflammation does not subside properly with steroid therapy, rethinking the diagnosis and considering the most relevant infectious etiologies is crucial. The pattern of occlusive retinal vasculitis in our patient was more leaned towards arteriolitis rather than phlebitis. This patient had diffuse retinal and choriocapillaris atrophy, but no active inflammation was noted in choroidal stroma or vessels. As soon as the diagnosis was made, anti-syphilitic therapy was initiated, which resulted in retinitis resolution and managed to salvage some extent of his peripheral visual field. The patient's cutaneous lesions were not typical of secondary syphilis skin manifestations, but their co-occurrence with the visual symptoms and immediate resolution after anti-syphilitic therapy brought us to consider them as atypical cutaneous manifestations of secondary syphilis. Several other atypical morphologies of secondary syphilis lesions have been reported as well. Among them are annular, nodular, pustular, acneiform, framboesiform, photosensitive SLE-like, corymbose, leukoderma, and pityriasis lichenoides-like morphologies^[9]^, many of which on their own in the absence of other diagnostic clues are not suggestive of syphilis.

The incidence of syphilis infection has increased considerably over the last decades^[1]^. To the best of our knowledge, this is the first reported case of ocular syphilis in Iran and denotes the importance of overcoming cultural limitations that exist in sexual history acquisition from patients with clinical patterns that raise suspicion of infectious diagnoses. Ophthalmologists should be cognizant of the various manifestations of this infection. Early treatment is time-sensitive in preventing permanent vision loss.

##  Conflicts of interest

None.

##  Funding 

None.
